# The Etiology and Pathological Anatomy of Aneurysm

**Published:** 1903-10

**Authors:** W. Lowndes Peple

**Affiliations:** Richmond, Va., Professor of Histology, University College of Medicine


ATLANTA

### Journal-Record of Medicine.

Successor to Atlanta Medical and Surgical Journal, Established 1855, and Southern Medical Record, Established 1870.

VOL. V. OCTOBER, 1903. No. 7.

BERNARD WOLFF, M.D., EDITOR, Nos. 319–20 Prudential.

Published Monthly.

M. B. HUTCHINS, M.D., BUSINESS MANAGER, 1007–1008 Century Bldg.

### ORIGINAL COMMUNICATIONS.

### THE ETIOLOGY AND PATHOLOGICAL ANATOMY OF ANEURYSM.*

BY W. LOWNDES PEPLE, M.D., RICHMOND, VA.,
Professor of Histology, University College of Medicine.

Before opening the subject of Aneurysm, let it be remembered that in spite of the various divisions and subdivisions of the wall of an artery, it is built of but two elementary tissues endowed, so far as our subject is concerned, with but three functions. The involuntary muscle lies in the middle coat and under the influence of the sympathetic nerves controls the vessel caliber, thus, under usual conditions, maintains a uniform blood-pressure. In the fibro-elastic tissues reside the other two functions. The yellow fiber is an elastic cushion upon which the force of the heart-beat falls, rendering an intermittent stream continuous. Of the white fibers is woven a resisting wall to stand guard lest elasticity be overtaxed or contractility should fail.

*Read before the Richmond Academy of Medicine and Surgery, July 28, 1903.

In the walls of the large arteries there is relatively little muscle and much elastic and fibrous tissues. As a consequence, in the variations of the general vascular capacity, these greater trunks bear but a small part.

In a paper of this scope, it is deemed best to avoid the discussion of the cirsoid aneurysm, the false aneurysm, the aneurysmal varix and the miliary cerebral aneurysm, giving the time to the common varieties met with in the larger vessels.

A true aneurysm is a permanent circumscribed dilatation of one or more coats of an artery. There are three varieties of aneurysm: the tubular or fusiform; the sacular and the dissecting aneurysm.

A tubular aneurysm consists of the dilatation of all the coats of an artery involving the entire circumference for a greater or less extent.

A sacular aneurysm is a sac-like dilatation upon the side of an artery or upon a tubular aneurysm. This never retains all three coats if the sac attains a considerable size.

A dissecting aneurysm is one in which the blood makes its way between the coats of the vessel, dissecting them apart and making there an abnormal space. This latter condition is rare.

*Aneurysm occurs in a previously diseased large artery of a man past forty who is subject to violent intermittent muscular exertion.* Aneurysm occurs in man because his occupations are more laborious than those of woman. It occurs at middle life because, while this is the age at which degenerative changes begin, he has not discontinued the heavy, laborious work of youth.

It occurs in a large vessel by reason of its structures, for the bulk of the wall is composed of connective tissue, which is especially prone to degenerative changes, and the muscular tissue which might supplement the elastic is here very scant. It occurs in a large vessel by reason of its anatomical location, for the great trunks not only bear the brunt of the heart-beat, but they also lack the sturdy support of muscle, bone and tendon enjoyed by vessels elsewhere.

The occurrence of aneurysm in a healthy vessel, unless one or more of its coats be mechanically severed, is so rare that it scarcely need be mentioned.

There are certain constitutional diseases and conditions, namely, syphilis, gout, rheumatism, alcoholism, etc., which are accompanied by changes within the vessel wall which may be said to precede aneurysm. These changes consist either of a softening of one or more of the coats, which allows dilatation to take place at the diseased area, or a hardening, which destroys the normal elasticity and, by throwing the strain elsewhere, gives rise to dilatation.

Atheroma, a rather vague term used to denote a local softening, seems to have for its direct cause the obliteration or plugging up of one of the vasa vasorum. There is first a round-cell proliferation in the intima, which forms a whitish patch and projects as an elevation in the lumen. Fatty degeneration and finally disintegration take place in the cells, and an atheromatous ulcer is formed.

Gumma, the tertiary syphilitic lesion, is followed by softening similar to atheroma.

Where isolated calcareous plates slough into the lumen a vulnerable point is left.

Arterio-sclerosis predisposes to aneurysm by destroying the elasticity of the vessel wall. It consists of a marked increase of fibrous tissue of both intima and adventitia and, to some extent, of the media, which undergoes more or less contraction. Robbed of its elasticity, the vessel wall gives way in some weak or less affected point.

Calcareous degeneration, when involving considerable areas, may give rise to aneurysm. Where the entire circumference is involved in a chalky cannula, for instance, there would be undue tension at the mouth of this tunnel. The causes enumerated may act singly or together. Syphilis, by many authors, is claimed to be a most fruitful source of aneurysm.

Of direct injury as a cause, it may be mentioned that with the advent of the modern high velocity small-caliber projectile, there will doubtless be more true traumatic aneurysm, for these bullets cut the outer coats like a knife and allow hernia of the inner to take place.

Microscopic section of an aneurysmal wall would, of course, vary with each case and would present the normal tissues greatly infiltrated and stretched, with external fibrous thickening of the adventitia, which is frequently amalgamated to all surrounding structures. The interior of the sac, especially if its orifice be small, contains a lamellated deposit of fibrin on the intima.

Having weakened the wall and provided an exciting cause, I now leave it to my colleague, who is to follow, to say whether or not my patient has developed aneurysm.

